# Account of Diseases in an Eastern District of London, from the 20th of May to the 20th of June, 1807

**Published:** 1807-07

**Authors:** 


### *Account of Diseases in an Eastern District of London, from the 20th of May to the 20th of June,* 1807.

| | |
|---|---|
| ACUTE DISEASES. | |
| Typhus - - - | 2 |
| Hydrocephalus Internus | 3 |
| Peripneumonia - | 2 |
| Hæmoptysis - - | 2 |
| Rheumatismus Acutus | 3 |
| CHRONIC DISEASES. | |
| Tussis - - - | 7 |
| Dyspnœa - - - | 3 |
| Tussis cum Dyspnœa - | 4 |
| Phthisis Pulmonalis - | 2 |
| Gastrodynia - - | 5 |
| Pyrosis - - - | 1 |
| Colica - - - | 1 |
| Vomitus - - - | 2 |
| Enterodynia - - | 5 |
| Diarrhœa - - - | 6 |
| Dysenteria - - | 1 |
| Menorrhagia - - | 4 |
| Dysmenorrhœa - | 3 |
| Chlorosis - - - | 6 |
| Leucorrhœa - - | 4 |
| Dysuria - - - | 2 |
| Vermes - - - | 1 |
| Rheumatismus Chronicus | 10 |
| PUERPERAL DISEASES. | |
| Menorrhagia Lochialis | 4 |
| Mastodynia - - | 3 |
| Rhagas Papillæ - | 5 |
| Hæmorrhois - - | 2 |
| INFANTILE DISEASES. | |
| Aphthæ - - - | 6 |
| Pertussis - - - | 3 |
| Tabes Mesenterica - | 2 |
| Vermes - - - | 3 |

Within a few weeks, three cases of hydrocephalus internus have occurred. This disease is often so insidious in its attack, and so secret in its progress, that it makes considerable advance before the nature of it is understood, or the the patient is thought sufficiently ill to need any particular medical attention. Children are almost the only subjects of it. The leading symptoms are uneasiness and pain in the head, which are discovered by the child's frequently applying its hand to the head, and, in some cases, suddenly screaming. Nausea and vomiting frequently succeed this symptom; and in the more advanced stages, a dilatation of the pupil of the eye, and occasional strabismus occur, and serve more strongly to mark the disease.—Towards the close a coma supervenes, and the patient dies in an apoplectic state. The disease has therefore by some nosologists been denominated apoplexia hydrocephalica. Cases of this kind generally prove fatal; though it is probable that, if the complaint were attended to when its first symptoms appear, and medical assistance were called in more early than it generally is, the mischief might be prevented.

In every case where there is a large head and a florid countenance, and where great sprightlyness and vivacity are discovered, we should consider the child as strongly predisposed to the disease.

With respect to the mode of cure, it will occur to every skilful practitioner, that a different treatment will be necessary in different stages of the disease. In the early period of it, the application of a number of leeches to the head may arrest its progress, and reduce the inflammation, with which it frequently commences. With the same intention cathartics may be employed, and the general antiphlogistic plan of treatment may be observed. In the advanced stages of the disease, mercurials may be administered in different forms, and in such a manner, as will best promote a discharge from the glands of the mouth.

